# A calculator based on prostate imaging reporting and data system version 2 (PI-RADS V2) is a promising prostate cancer predictor

**DOI:** 10.1038/s41598-019-43427-9

**Published:** 2019-05-03

**Authors:** Hui Wang, Sheng Tai, Li Zhang, Jun Zhou, Chaozhao Liang

**Affiliations:** 1 Department of Urology, The First Affiliated Hospital of Anhui Medical University, Hefei, China; 2 The institute of Urology, Anhui Medical University, Hefei, China; 3 Anhui Province Key Laboratory of Genitourinary Diseases, Anhui Medical University, Hefei, China

**Keywords:** Prostate cancer, Prostate

## Abstract

This research is to develop a new tool to improve the performance of predicting prostate cancer (PCa) and reducing unnecessary biopsies. The clinical data of patients who were definitely diagnosed by prostate biopsy were retrospectively analyzed. PCa risks that include age, prostate-specific antigen (PSA), PSA density (PSAD), free-PSA (fPSA), the ratio of fPSA to PSA (%fPSA), prostate volume (PV), digital rectal examination (DRE) and multi-parametric magnetic resonance imaging (MP-MRI) were selected by univariate and multivariate analysis. The satisfactory risks were used to establish predictor (Prostate Biopsy Rating Scale, PBRS). The total score (TS) that was obtained from PBRS was performed to forecast PCa. The areas under the receiver operating characteristic curve (AUC) and the net reclassification index (NRI) were used to compare the predictive ability. A total of 1078 cases were recruited. The mean values of TS in PCa and non-PCa were 15.94 ± 3.26 and 10.49 ± 3.36 points respectively. The AUC of PBRS was higher than PSA, PSAD and MP-MRI (0.87 vs. 0.75, 0.78, 0.80, respectively). PBRS can reduce unnecessary biopsies compared with PSA, PSAD and MP-MRI by up to 63%, 54% and 44%, respectively. In brief, PBRS is a promising predictor of forecasting PCa.

## Introduction

Urologists sometimes encountered the situation that patients with the normal test of multi-parametric magnetic resonance imaging (MP-MRI) had an elevated level of prostate-specific antigen (PSA), or patients with normal PSA level existed an abnormal result on MP-MRI. As a urologist, what are the next treatment opinions? According to the report on Cancer Surveillance Center, the incidence rate of prostate cancer (PCa) is continually rising^1,2^. PCa, in China, has grown up to be a danger to the men’s health, and its incidence rate is increasing year after year with generalizing the PSA screening^3^. At present, Chinese Guidance for Diagnosis and Treatment of Urology still suggests that PSA is the primary indicator of screening PCa due to the convenience and low economic costs^3^.

The widespread PSA screening can increase the detection rate and reduce the mortality rate, but many factors have an influence on PSA level, including age, prostate volume (PV), digital rectal examination (DRE) and so on. Therefore, these risks should be taken into account in screening PCa^4–7^. Furthermore, there were reports suggested that prostate imaging reporting and data system version 2 (PI-RADS v2) is an excellent measure to predict PCa compared with PSA/PSAD^8–10^ and has better performance in identifying patients with clinically significant PCa^11^. Based on the probability, PI-RADS can divide the screening crowd into five groups, which used 1 to 5 points to represent the probability of suffering from PCa: highly unlikely (1 point), unlikely (2 points), equivocally (3 points), likely (4 points) and highly likely (5 points). In this research, we, based on PI-RADS v2, try to develop a new tool to predict PCa.

## Results

### Patient demographics

Overall, there were 544 men with PCa (51%) and 534 men without PCa (49%). The incidence of PCa increased with the increase of age, PSA, PSAD, PI-RADS and TS, and increased with the decrease of PV, details in Table 1. The distribution of demographics in different PSA, Gleason score, clinical stage and D’Amico risk were also exhibited (Supplementary Table 1). The biopsy cores for mean in all patients, healthy patients and PCa patients were 12.15, 12.38 and 11.92, respectively (Supplementary Table 1).

**Table 1:** The clinical characteristics and demographics of all.

Characteristic	Training cohort (N = 1078)				Validation cohort (N = 178)		
	Total	Non-PCa N = 534 (49%)	PCa N = 544 (51%)	P	Non-PCa N = 86 (48%)	PCa N = 92 (52%)	P
**Mean**(**SD**)							
PBRS (total score)	13.24 (4.29)	10.49 (3.36)	15.94 (3.26)	<0.001	10.74 (3.11)	16.14 (3.65)	<0.001
Age, years	68.90 (8.28)	66.85 (8.22)	70.91 (7.85)	<0.001	68.23 (7.42)	71.01 (8.24)	0.002
PSA level, ng/ml	30.95 (10.90)	17.90 (8.31)	43.76 (15.11)	<0.001	16.15 (16.59)	46.86 (38.99)	<0.001
fPSA, ng/ml	6.59 (11.49)	3.49 (6.73)	9.62 (14.10)	<0.001	3 (6.44)	11.42 (16.13)	<0.001
%fPSA	0.22 (1.52)	0.17 (0.13)	0.26 (2.41)	0.32	0.17 (0.09)	0.19 (0.15)	0.176
PSAD, ng/ml/ml	0.74 (0.90)	0.40 (0.51)	1.07 (1.07)	<0.001	0.27 (0.3)	1.01 (1.02)	<0.001
PV, ml	53.87 (36.22)	58.76 (36.15)	49.08 (35.68)	0.001	66.85 (26.35)	54.85 (38)	0.008
Erythrocyte	4.46 (0.59)	4.52 (0.56)	4.4 (0.61)	0.006	4.52 (0.66)	4.36 (0.65)	0.115
Platelets	196.48 (65.02)	201.17 (69.11)	191.09 (59.89)	0.031	213.58 (67.23)	187.83 (65.35)	0.01
Neutrophil	4.06 (3.41)	4.09 (1.78)	4 (4.46)	0.891	4.15 (1.92)	3.88 (1.33)	0.285
Lymphocyte	1.73 (2.3)	1.66 (0.73)	1.8 (3.15)	0.482	1.65 (0.72)	1.53 (0.54)	0.195
NLR	2.88 (2.58)	2.91 (2.22)	2.85 (2.88)	0.780	3.11 (2.99)	2.86 (1.59)	0.513
BMI	23.35 (3.37)	23.47 (3.05)	23.24 (3.66)	0.283	22.76 (3.12)	22.88 (3.21)	0.307
**PI-RADS v2**							
1–2	224 (21)	196 (37)	28 (5)	<0.001	36 (41)	5 (5)	<0.001
3	331 (30)	208 (39)	123 (23)	<0.001	31 (36)	19 (21)	<0.001
4	306 (29)	114 (21)	192 (35)	<0.001	16 (19)	36 (39)	<0.001
5	217 (20)	16 (3)	201 (37)	<0.001	3 (4)	32 (35)	<0.001

Abbreviation: PCa: prostate cancer; SD: standard deviation; PBRS: prostate biopsy rating scale; fPSA: free prostate-specific antigen; %fPSA: fPSA to PSA ratio; PSAD: PSA density; PV: prostate volume; NLR: neutrophil to lymphocyte ratio; BMI: body mass index; PI-RADS V2: prostate imaging reporting and data system version 2.

### Prostate biopsy rating scale (PBRS)

All associated risks were analyzed by univariate and multivariate analysis. We selected the satisfactory risks to develop the PBRS, including age, PSA, PSAD, PV and MP-MRI. DRE, fPSA, %fPSA, BMI, erythrocyte, neutrophil, platelets and NLR were excluded from our research (details presented in Tables 1 and 2).

**Table 2:** The results of multivariate stepwise logistic regression.

Indicators	B	S.E	Wals value	P	OR (95%CI)
Age	0.08	0.01	59.88	0.00	1.09 (1.06–1.11)
PSA	0.04	0.01	43.47	0.00	1.05 (1.03–1.06)
fPSA	−0.03	0.01	2.90	0.093	0.98 (0.95–1.00)
PSAD	0.12	0.25	0.23	0.00	1.13 (0.70–1.83)
PV	−0.01	0.00	8.18	0.00	0.99 (0.99–1.00)
MP-MRI	1.07	0.09	145.01	0.00	2.92 (2.45–3.47)
Erythrocyte	0.086	0.176	0.239	0.233	1.09 (1.06–1.11)
Platelets	−0.04	0.02	3.31	0.321	1.00 (0.96–1.00)

Abbreviation: fPSA: free prostate-specific antigen; PSAD: PSA density; PV: prostate volume; MP-MRI: multi-parametric magnetic resonance imaging; OR: odds ratio. SE: standard error; B: represent the coefficient of equation.

Based on equation coefficient and odds ratio (Exp (B)), the weight coefficient ratio of age, PSA, PSAD, PV and MP-MRI in developing model was 1: 1: 1: 1: 3, respectively. So, the point of PI-RADS is three times that of other risks. The probability intervals of PI-RADS respectively were ≤15%, 15–40%, 40–65% and ≥65%, which respectively corresponded to points of PI-RADS: 1–2 points, 3 points, 4 points and 5 points. The mean and 95% CI of all risks in above four probability intervals were calculated (Table 3 and Supplementary Table 2). The upper bound of 95% CI was regarded as the threshold among groups (Table 3). Each patient can get a TS ranging from 0 to 21 points, and the screening threshold of TS was 13 points or higher.

**Table 3:** The regression probability of MRI in predicting PCa and the thresholds of each group constituted the prostate biopsy rating scale (PBRS).

Indicators	0 point	1 point	2 points	3 points
	Unlikely (normal)	Equivocally	Likely	Highly likely
**MP-MRI**				
PI-RADS v2	1–2	3	4	5
Regression probability	≤0.15	0.15–0.40	0.40–0.65	>0.65
**Thresholds**				
Main indicators				
PSA (ng/ml)	0–4	4–10	10–26	>26
PSAD (ng/ml^2^)	≤0.15	0.16–0.20	0.20–0.60	>0.60
PI-RADS (MP-MRI)*	1–2	3	4	5
**Auxiliary indicators**				
Age (year)	<50	50–58	59–70	>70
PV (ml)	>454	232–454	42–232	<42

Notes: 1. Total score (TS): 0–21 points; Negative screening: <12 points, Positive screening: ≥13 points. 2. ^*^This group points are three times that of other groups.

Abbreviations: PSA: prostate-specific antigen; PSAD: PSA density; PV: prostate volume; MP-MRI: multi-parametric magnetic resonance imaging; CI: confidence interval.

### Comparison between PBRS and other indicators

**Figure 1:**
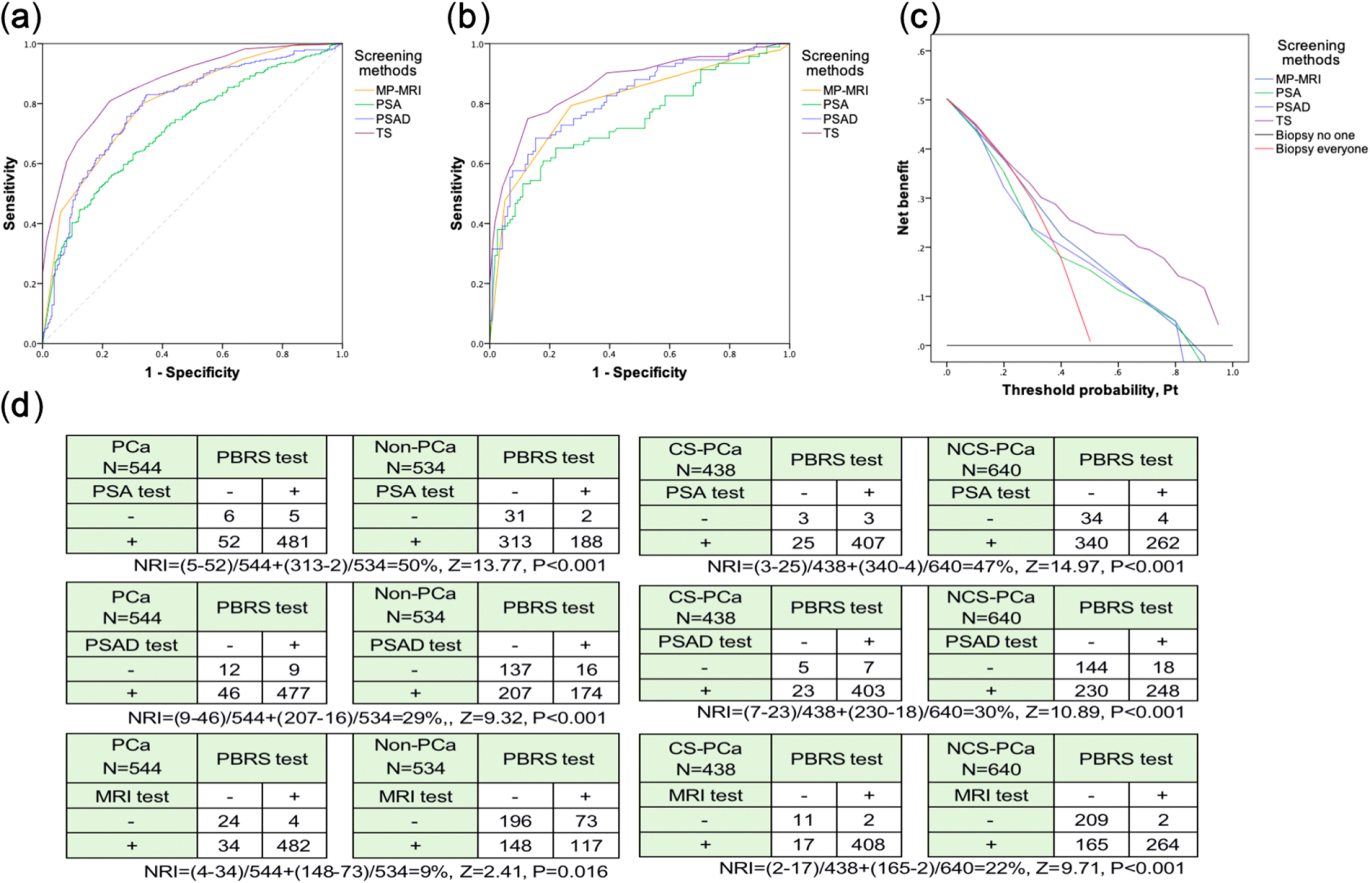
ROC curves, decision curve analysis (DCA) and net reclassification index (NRI) were used to show the ability of predicting PCa. (**a**) ROC curves were used to illustrate the performance of forecasting PCa in the training cohort (N = 1078). (**b**) Validating the performance of screening PCa in the validation cohort (N = 178) by ROC curves. (**c**) DCA was displayed to reveal the net benefit in different threshold probability. (**d**) NRI can make clear the ability that each indicator correctly reclassifies patients. The value of NRI was proportional to the ability of reclassifying. ROC = receiver operating characteristic curve; PCa = prostate cancer; CS-PCa = clinically significant PCa.

The AUC of PBRS, PSA, PSAD and MP-MRI were 0.87, 0.75, 0.79 and 0.80 respectively, P < 0.01 (Fig. 1(a) and Table 4). Most important is that the AUC of PBRS always was the highest in the different PSA level (Table 5). DCA was applied to evaluate the performance of predicting PCa (Fig. 1(c)). PBRS had greater net benefit than individual indicators at any probability. PSA and PSAD were of no additional net benefit until the threshold probability (PT) was close to 25–30%, and the additional net benefit disappeared when threshold reached 70%. In contrast, PBRS began to show benefit when PT was close to 20%, and PBRS had the best benefit when PT was over 20%.

**Table 4:** Evaluation indexes were used to explicate the ability of four indicators in forecasting PCa.

Evaluation indexes	PSA	PSAD	MP-MRI	PBRS
Youden’s index	0.04	0.25	0.32	0.57
Likelihood ratio (+)	12.41	135.35	179.02	356.39
PPV (%)	533/1034 (52)	523/904 (58)	516/854 (60)	445/586 (76)
OR (95%CI)	3.19 (1.60–6.38)	10.00 (6.22–16.08)	10.69 (7.03–16.25)	12.69 (9.48–16.98)
Overall accuracy (%)	566/1078 (53)	676/1078 (63)	712/1078 (66)	839/1078 (78)
AUC (95%CI) for all cancer	0.75 (0.72–0.78)	0.79 (0.76–0.81)	0.80 (0.78–0.83)	0.87 (0.85–0.89)
AUC (95%CI) for Gleason score >=7	0.77 (0.74–0.80)	0.79 (0.77–0.82)	0.81 (0.79–0.84)	0.87 (0.85–0.89)
AUC (95%CI) for Clinical stage >=T2b	0.79 (0.76–0.82)	0.79 (0.77–0.83)	0.80 (0.77–0.83)	0.86 (0.84–0.89)
AUC (95%CI) for D’Amico risk (>Low)	0.79 (0.76–0.81)	0.81 (0.79–0.84)	0.81 (0.79–0.84)	0.88 (0.86–0.90)

Abbreviations: PCa: prostate cancer; PSA: prostate-specific antigen; PSAD: PSA density; MP-MRI: multi-parametric magnetic resonance imaging; CI: confidence interval. PBRS: prostate biopsy rating scale; PPV: positive predictive value; OR: odds ratio; AUC: area under the curve.

**Table 5:** The four predictors’ performance of predicting PCa at different ages and PSA values were shown by AUCs and overall accuracy.

Characteristic	AUC (95%CI)				Overall accuracy			
	PSA	PSAD	MP-MRI	PBRS	PSA	PSAD	MP-MRI	PBRS
**Age (years)**								
<=55	0.73 (0.61–0.86)	0.77 (0.65–0.89)	0.89 (0.82–0.97)	0.91 (0.84–0.98)	0.41	0.41	0.51	0.77
56–65	0.79 (0.73–0.84)	0.84 (0.79–0.89)	0.83 (0.78–0.88)	0.89 (0.85–0.93)	0.43	0.55	0.62	0.79
66–75	0.75 (0.7–0.79)	0.79 (0.75–0.83)	0.78 (0.74–0.82)	0.83 (0.8–0.87)	0.51	0.63	0.64	0.73
>75	0.71 (0.65–0.78)	0.77 (0.7–0.84)	0.84 (0.78–0.89)	0.88 (0.84–0.93)	0.70	0.78	0.79	0.83
**PSA (ng/ml)**								
0–4	0.45 (0.25–0.66)	0.47 (0.25–0.69)	0.88 (0.77–0.99)	0.91 (0.83–0.99)	0.75	0.66	0.45	0.82
4–10	0.61 (0.52–0.69)	0.72 (0.64–0.79)	0.74 (0.67–0.81)	0.83 (0.78–0.89)	0.26	0.59	0.52	0.77
10–20	0.55 (0.49–0.62)	0.72 (0.67–0.78)	0.79 (0.74–0.83)	0.83 (0.79–0.88)	0.42	0.52	0.61	0.71
20–30	0.53 (0.43–0.63)	0.70 (0.61–0.79)	0.77 (0.69–0.85)	0.80 (0.72–0.87)	0.53	0.55	0.68	0.72
30–40	0.65 (0.51–0.80)	0.65 (0.51–0.79)	0.72 (0.58–0.86)	0.76 (0.63–0.89)	0.65	0.65	0.75	0.78
>40	0.67 (0.59–0.76)	0.59 (0.50–0.68)	0.82 (0.76–0.88)	0.84 (0.79–0.90)	0.82	0.82	0.85	0.85

Abbreviations: PSA: prostate-specific antigen; PSAD: PSA density; MP-MRI: multi-parametric magnetic resonance imaging; CI: confidence interval. PBRS: prostate biopsy rating scale; AUC: area under the curve.

**Figure 2:**
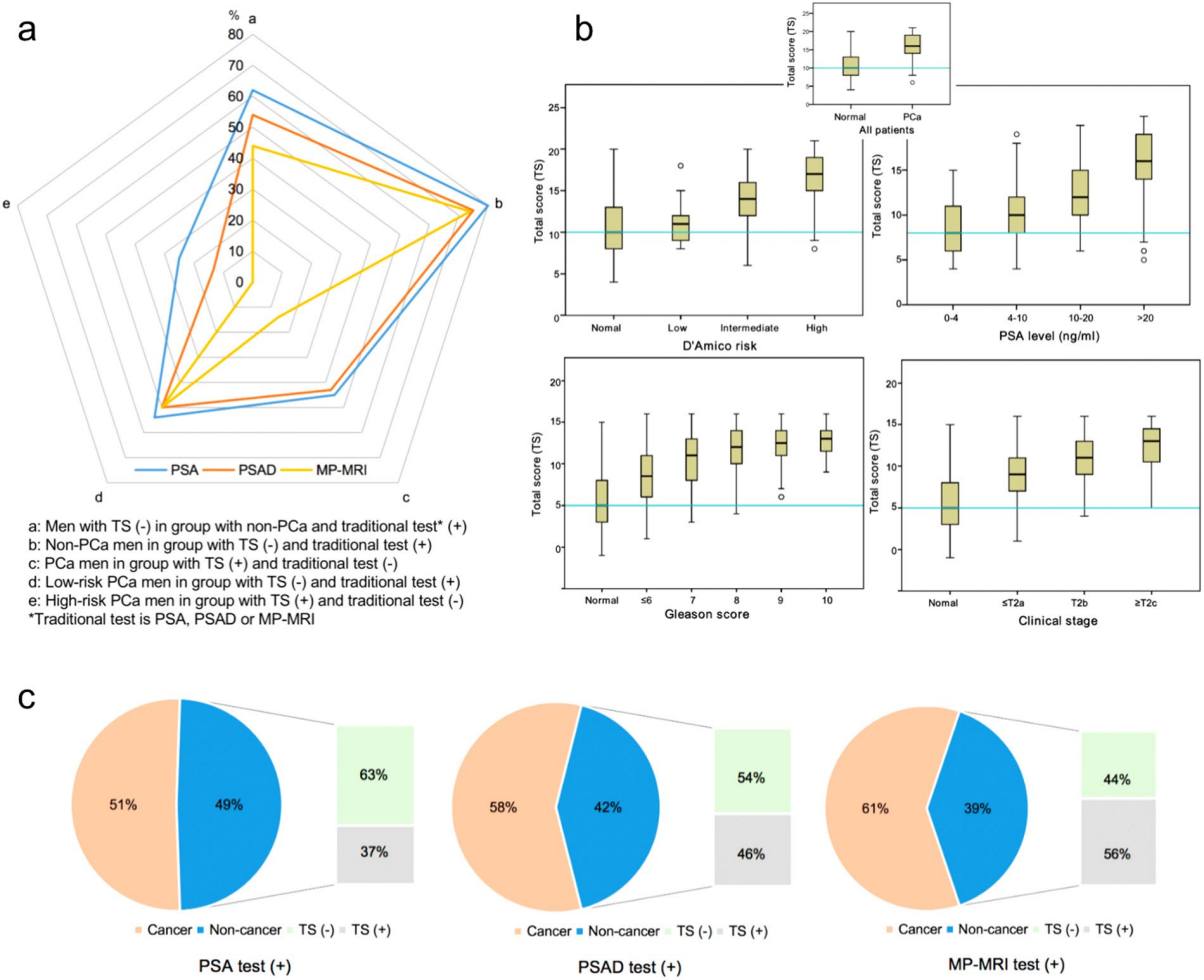
The distribution of total score in the different cohorts and the illustration about the reduction of unnecessary biopsies in the process of predicting PCa by PBRS. (**a**) PBRS can reduce the number of unnecessary biopsies in the cohort with different characteristics. The proportion of non-PCa patients who were positively tested by other indexes reduced by applying PBRS or the number of PCa patients who were negatively tested by other indexes increased by using PBRS. (**b**) The box-plot was used to show that the value of total score is highly associated with PCa and the grades of PCa. (**c**) Many patients who were positively tested by other indexes were not with PCa actually, which can be correctly predicted by PBRS in most time so that can prevent these patients from the biopsy. PCa = prostate cancer; PBRS = prostate biopsy rating scale.

We discovered that PBRS had a higher Youden’s index (sensitivity + specificity −1), PPV, OR, likelihood ratio than other predictors. For Youden’s index, PBRS was 14, 2.3 and 1.8 times of PSA, PSAD and MRI, respectively (Table 4). Besides, the percentage of correct classification was calculated. We noted that PBRS still had the highest value, especially in the group of low age (<75 years) and group of PSA <40 ng/ml. But, with the increase of age and PSA, the advantage gap gradually narrowed until it disappeared (Table 5). Besides, the distribution of TS in the different groups was represented by box-plot (Fig. 2(b)).

### Reduction of unnecessary biopsies

PSA >4 ng/ml, PSAD >0.15 ng/ml^2^, MP-MRI >2 and TS ≥13 were regarded as thresholds between the negative and positive group. We counted the proportion of patients with the following situations: (1) patients with negative TS test in cohort that were diagnosed with non-PCa by biopsy but had positive findings on PSA, PSAD or MRI; (2) patients with non-PCa in cohort that had negative test on TS but had positive tests on PSA, PSAD or MRI; (3) patients with PCa in cohort that had positive test on TS but had negative tests on PSA, PSAD or MRI; (4) patients with low-risk PCa in cohort that had negative test on TS but had positive tests on PSA, PSAD or MRI; (5) patients with high-risk PCa in cohort that had positive test on TS but had negative tests on PSA, PSAD or MRI. These situations were shown in Fig. 2(a). The above results suggested that TS not only had a better performance in predicting PCa but had a better ability in identifying patients with high-risk PCa. In order to illustrate the significance of PBRS in reducing unnecessary biopsies, we used sector graph to show these results (Fig. 2(c)). As can be seen from Fig. 2(c), we can know that PBRS can respectively prevent 63%, 54% and 44% of normal men from undergoing biopsy compared with PSA, PSAD and MP-MRI.

### Validation and calibration

The validation cohort was used to validate the performance of PBRS in predicting PCa externally. The results revealed that PBRS has a stable and excellent ability in forecasting PCa. The ROC was shown in Fig. 1(b). The overall accuracy of PBRS in validation cohort was 81%, and the sensitivity and specificity respectively were 0.73 and 0.88. However, for PSA and PI-RADS, the overall accuracy respectively was 56% and 72%, and the sensitivity and specificity respectively were 0.46 and 0.85 for PSA and 0.70 and 0.73 for PI-RADS.

**Figure 3:**
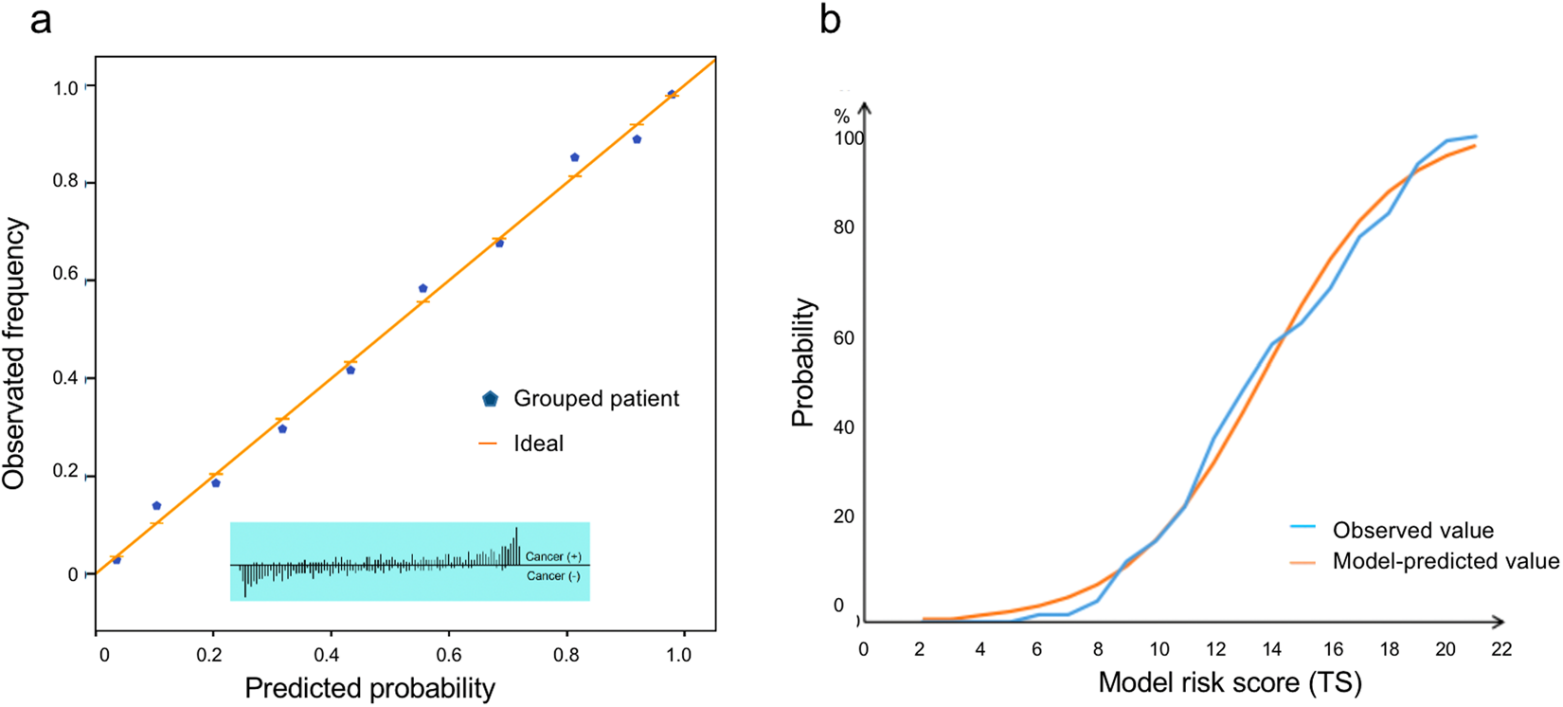
The discrimination and calibration of each indicator. (**a**) Calibration curves for the predictive models and the distribution of the frequency of patients in the different predicted probabilities were shown at the bottom of the graphs, separating those with (+) and without (−) cancer. (**b**) The goodness of fit between observed and predicted probability with the total score increasing.

In addition, we used calibration curves and the curves of goodness of fit to exhibit the predictive discrimination and calibration (Fig. 3(a,b)). PBRS underestimated the probability of occurring PCa when TS was between 11 and 15 points and when TS was higher than 19 points. Hosmer-Lemeshow test was used to calculate the value of calibration (X^2^ = 12.07, P = 0.708). Besides, the net reclassification index (NRI) was estimated to evaluate the ability of reclassification. Compared with PSA, PSAD and MP-MRI, PBRS can respectively make 50%, 29% and 9% patients exactly reclassify (Fig. 1(d)), (P < 0.01). In addition, for the clinically significant PCa, the NRIs reached 47%, 30% and 22%, respectively, P < 0.001.

## Discussion

At present, many calculators can be used to predict PCa, such as nomogram, Prostate Cancer Prevention Trial risk calculator (PCPT-RC), European Randomized Study for Screening of Prostate Cancer (ERSPC-RC) and so on. However, these calculators had several limitations. First, parts of calculators used DRE instead of PI-RADS. Regrettably, DRE cannot quantify the probability of occurring PCa, and can only be qualitatively described, which could decrease the ability in predicting PCa if DRE is used to develop the predictive model. Second, although these methods combined multiple indicators, they were not very convenient to use. Sometimes only with the help of computer programs, the user can use these methods to predict PCa. In contrary, when you are evaluating the probability of suffering from PCa by PBRS, you need to calculate TS based on the results of each examination.

Now, some studies have shown that novel biochemical markers (such as PSA precursors, PCa antigen 3, etc.) were superior to PSA in the detection of PCa^12–14^. But, parts of these are still in the period of exploration and validation, so that it has not been widely used to screen PCa in the clinic, especially in China. In this sense, PSA is still the most important and economical biochemical immune index to screen PCa. Currently, MP-MRI, in China, is difficult to be executed to screen PCa on a large scale due to the time and economic costs. Many studies had suggested that PI-RADS v2 had excellent ability in predicting PCa, especially for clinically significant PCa^4,11,15,16^. More and more patients with low-risk PCa were founded via PSA or MP-MRI, which may cause over-treatment. Therefore, a predictive model needs a better ability to identify the intermediate or high-risk PCa. Our study based on PI-RADS v2 can significantly increase the capacity of predicting PCa, and also had outstanding performance in identifying intermediate-risk or high-risk PCa. With these advantages, PBRS not only can reduce the number of unnecessary biopsies but also can decrease the amount of over-treatment for low-risk PCa. There were some researches revealed that MP-MRI combined PSA or PSAD can improve the ability for PCa detection compared with PSA and PSAD alone^15,17,18^, but the individualized indexes (such as age, PV) were not included in these researches, and most important is that these studies didn’t add PI-RADS to the predictive model.

It is essential to matter that we should be aware of prerequisites before using PBRS to predict PCa. The use of PBRS to evaluate the likelihood of having PCa is primarily for patients who had abnormal findings on PSA, MRI or DRE. We don’t suggest that PBRS is widely used to screen PCa for everyone. Besides, PBRS consisted of two parts, including main indicators and auxiliary indicators. Age > 70 years, PV < 42 ml, PSA >26 ng/ml and 5 points of PI-RADS obtained same points (3 points), which doesn’t mean that those indicators had same ability in solely predicting PCa, but it means that age, PV, PSA or PI-RADS can provide same reference value to each other in predicting PCa. As we all know, in the same conditions of PSA, PSAD and MP-MRI, men with higher age and smaller PV maybe have a higher probability of having PCa. So, age and PV are essential reference indexes in the detection of PCa for men with a positive test on PSA, PSAD, DRE or MP-MRI. Last but not least, in the process of screening PCa, PBRS must be utilized as a whole rather than be separated or be chosen partially to apply.

PBRS provides a novel method for urologists to predict PCa, which contribute to the decrease of over-treatment and unnecessary biopsies by up to a maximum of 63%. These advantages not only were exhibited in predicting PCa but also were shown in identifying the intermediate-risk and high-risk PCa. Regrettably, we noted that the superiority gap between PBRS and other indicators in predicting PCa narrowed with the increase of age, PSA value and PI-RADS points. Even so, PBRS still had the best performance in screening PCa. This gap disappeared between MP-MRI and PBRS when patients were over 75 years old or the PSA value is over 40 ng/ml, which might indicate that PBRS may work best at a lower age and PSA value.

In addition, PBRS can correctly make more people reclassify, which means that more people with PCa can be accurately predicted and more people without PCa can be excluded from a cohort that needs to suffer from biopsy. At present, an increasing number of studies suggested that men with clinically significant PCa (Gleason score 3 + 4 score or higher) need to treat, so even if the low-risk PCa is discovered, it does not require treatment immediately, but require active surveillance. From our results, we found that the mean (95% CI) of TS for men who had the Gleason score 3 + 3 or lower was 13.55 (12.9 to 14.19), and for men with the clinically significant PCa was 16.10 (15.1 to 17.27). Therefore, men whose TS is 13 or 14 maybe suffer from low-risk PCa so that these people could take active surveillance instead of immediate treatment.

Finally, several limitations of our study need to be noted. First, the study was a retrospective study. Second, some people with PSA value ranging from 4 to 10 ng/ml refused to receive biopsy and preferred to choose follow-up observation, which reduced the number of these people in this study. Third, men were diagnosed with PCa through pathology obtained by TRUS biopsy that also exists false positive and false negative, so that the error of biopsy itself can also make our results bias^19^. Therefore, the findings of PBRS need to be verified in the future by a prospective study with multicenter and a larger sample size.

In a word, PBRS makes the multiple indicators into a simple scale, which not only improves the ability for the detection of PCa and increases the proportion of correct classification but also it is more convenient to be used by urologists. So, PBRS is a promising tool for PCa detection.

## Methods

### Patients

Patients with the abnormal screening findings, such as MP-MRI, PSA, PSAD DRE and so on, were subsequently undergoing the trans-rectal ultrasound (TURS) guided 12 + X-core prostate biopsy (PB) in our cancer center. We retrospectively collected the clinical data in the electronic hospital system for these patients between January 2015 and June 2018 to establish the predictive model (n = 1235). In addition, we also gathered the clinical data for patients who underwent PB between July 2018 and December 2018 to externally validate the developed model (n = 194). TURS guided 12 + X-core prostate biopsy was conducted under the reports described by Presti JR^20,21^.

Some patients were excluded from this research due to the insufficient data, the details were as follow in the training group (n = 157, 12%): MP-MRI (n = 91), fPSA (n = 42), and the vague report of pathology (n = 24), and in the validation group (n = 16, 8%): MP-MRI (n = 3), prostate volume (n = 6), DRE (n = 5) and the vague report of pathology (n = 2). Eventually, there were 1078 patients in the training group and 178 patients in the validation group.

A standard protocol of this study was approved by Ethics Committees Regarding Human Research of the First Affiliated Hospital of Anhui Medical University (Approved ID: PJ-20170906). All experiments were performed following the relevant guidelines of the Institutional Ethics Committee and the Helsinki Declaration. This study was retrospective research, which just collected previous data from hospital system and didn’t include the use of tissue samples and other samples, and the study did not include any personal information or privacy. For these reasons, it was not applicable to the informed consent of study participators, which was also exempted by the Ethics Committee.

### Risks collection and PBRS development

The risks that are related with PCa were recommended by the Chinese Guidance for Diagnosis and Treatment of Urology and other previously published reports, and these risks were reconfirmed by the experienced chief physicians and professors of our institute. In this research, age, PSA, PSAD, fPSA, ratio of fPSA and PSA (%fPSA), DRE, MP-MRI, body mass index (BMI), erythrocyte, neutrophil, platelets and neutrophil to lymphocyte ratio (NLR) were involved.

First of all, we used univariate analysis to analyze the above risks to obtain risks that are associated with PCa. In other words, the obtained risks had statistical significance between PCa and non-PCa. These risks subsequently were evaluated by multivariate stepwise regression analysis to acquire satisfactory risks that are the independent risk factors of PCa. The original values of all satisfactory risks were standardized by logarithmic transformation. Then, the standardized risks were analyzed by logistic regression to calculate odds ratio (OR) and equation coefficients in the predictive model, which determined the weight coefficient of each risk in model development.

The five grades of PI-RADS (1 to 5 points) in evaluating PCa were converted to five probability intervals of predicting PCa. The probabilities of all satisfactory risks in predicting PCa were calculated, then, all risks were divided into five groups relying on the five probability intervals of PI-RADS. The mean and its 95% confidence interval (CI) of original values of all satisfactory risks whose probabilities located on the above five probability intervals were calculated, and the upper bound of 95% CI was regarded as the boundary among groups. Finally, the total score (TS) of prostate biopsy rating scale (PBRS) for each patient is the sum of the product of indicator’s score and weight coefficient. A flowchart showed details (Supplementary Fig. 1).

### Category and thresholds

According to the D’Amico risk classification^22^, PCa with PSA < 10 ng/ml, Gleason score < 7 and clinical stage < T2b is regarded as low-risk PCa. We separated patients into two groups based on pathology: PCa and non-PCa. In addition, patients, based on the screening thresholds of predictive indicators, also were divided into negative test group and positive test group. The thresholds of a positive test are as follows: PSA > 4 ng/ml, PI-RADS > 2 points, PSAD > 0.15 ng/ml^2^ and TS > 12.

### Statistical analysis

Univariate and multivariate analysis were used to filter out indicators. We used logistic regression to calculate the probabilities of predicting PCa and equation coefficients. Comparing the performance of detecting PCa, we calculated the Youden’s index, positive predictive value (PPV) and overall accuracy relying on the thresholds. In addition, the receiver operating characteristic curve (ROC) and decision curve analysis (DCA) generated by A.Vickers were also evaluated and created^23,24^. The maximal point of the sum of sensitivity and specificity determined the optimal threshold. Net reclassification index (NRI) was calculated and was tested by Z-test. Hosmer-Lemeshow goodness-of-fit test was applied to test the goodness of fit, and we used the calibration with 1000 bootstrap samples to decrease the over-fit bias. The statistical analysis was performed with SPSS version 23.0 and R package version 3.0. (https://www.r-project.org). P < 0.05 was considered as statistical significance.

## Supplementary information

Supplementary tables and figures

## Data Availability

All data generated or analyzed during this study are included in this published article (and its Supplementary Information files).
