# Supplementary material for: A calculator based on prostate imaging reporting and data system version 2 (PI-RADS V2) is a promising prostate cancer predictor
Source: Sci Rep. 2019 May 3;9:6870. doi: 10.1038/s41598-019-43427-9 (PMC6499813; doi:10.1038/s41598-019-43427-9)

A calculator based on prostate imaging reporting and data system version 2 (PI-RADS V2) is a promising prostate cancer predictor

Hui Wang[1,2,3], Sheng Tai[1,3], Li Zhang[2,3], Jun Zhou[1,2,3], Chaozhao Liang[1,2,3*]

[1]Department of Urology, the First Affiliated Hospital of Anhui Medical University, Hefei, China.

[2]The institute of Urology, Anhui Medical University, Hefei, China.

[3]Anhui Province Key Laboratory of Genitourinary Diseases, Anhui Medical University, Hefei, China.

***Correspondence**: Chaozhao Liang; Department of Urology, the First Affiliated Hospital of Anhui Medical University, Hefei 230022, China; Institute of Urology, Anhui Medical University, Hefei 230022, China.

Location: No. 218 Jixi Road, Shushan District, Hefei, China.

Tel: +86-0551-6292-3440

Fax: +86-551-63633742

E-mail: liang_chaozhao@ahmu.edu.cn

Supplementary Table 1. Demographics and biopsy findings of all men in different cohorts.

Characteristic	Total	Non-PCa	PCa (D'Amico risk)			
			Total	Low	Intermediate	High
N (%)	1078	534 (49)	544 (51)	28 (5)	113 (21)	403 (74)
PSA (ng/ml)						
0-4	44 (4)	33 (75)	11 (25)	4 (36)	4 (36)	3 (28)
4-10	237 (22)	176 (74)	61 (26)	24 (39)	23 (38)	14 (23)
10-20	329 (31)	192 (58)	137 (42)	0	86 (63)	51 (37)
>20	468 (43)	133 (28)	335 (72)	0	0	335 (100)
Gleason score						
<=6	107 (20)	-	107 (20)	28 (27)	39 (36)	40 (37)
7	160 (29)	-	160 (29)	0	74 (46)	86 (54)
>=8	277 (51)	-	277 (51)	0	0	277 (69)
Clinical stage						
<=T2a	146 (27)	-	146 (27)	28 (19)	58 (40)	60 (41)
T2b	155 (29)	-	155 (29)	0	55 (36)	100 (64)
>=T2c	243 (44)	-	243 (44)	0	0	243 (100)
Total biopsy cores (positive cores)	12.15	12.38	11.92 (6.6)	12.25 (2.36)	12.50 (4.10)	11.73 (7.60)

Abbreviation: Non-PCa: non prostate cancer; PSA: prostate-specific antigen.

Supplementary Table 2. The mean and 95% CI of the values of PSA, PSAD, MP-MRI and PBRS in different groups.

Characteristic	Mean (95% CI)			
	PSA	PSAD	MP-MRI	PBRS (TS)
Regression probability				
0.15-0.40	9.36 (9.05-9.67)	0.19 (0.19-0.20)	3	-
0.41-0.65	25.69 (24.85-26.53)	0.60 (0.58-0.61)	4	-
>0.65	82.06 (79.43-84.69)	2.06 (1.93-2.20)	5	-
Gleason score				
<=6	26.40 (21.44-31.35)	0.65 (0.53-0.78)	3.45 (3.25-3.65)	13.55 (12.9-14.19)
7	39.72 (34.35-45.08)	0.92 (0.79-1.05)	3.96 (3.82-4.1)	15.58 (15.1-16.06)
>=8	52.72 (48.48-56.96)	1.31 (1.16-1.45)	4.27 (4.18-4.37)	16.94 (16.61-17.27)
Clinical stage				
<=T2a	25.76 (21.38-30.15)	0.67 (0.56-0.78)	3.61 (3.44-3.78)	13.98 (13.45-14.51)
T2b	41.02 (35.31-46.72)	1.09 (0.86-1.32)	3.97 (3.83-4.11)	15.69 (15.2-16.17)
>=T2c	56.35 (52.04-60.66)	1.29 (1.17-1.4)	4.30 (4.19-4.41)	17.14 (16.79-17.5)
D'Amico risk				
Low	7.07 (6.15-7.98)	0.23 (0.18-0.27)	3.25 (2.83-3.67)	11.46 (10.45-12.48)
Intermediate	12.77 (11.98-13.57)	0.39 (0.35-0.42)	3.71 (3.53-3.88)	13.96 (13.42-14.51)
High	55.05 (51.69-58.4)	1.32 (1.21-1.43)	4.16 (4.08-4.25)	16.73 (16.44-17.01)

Abbreviation: PSA: prostate-specific antigen, PSAD: PSA density, PV: prostate volume, MP-MRI: multi-parametric magnetic resonance imaging, CI: confidence interval. PBRS: prostate biopsy rating scale, TS: total score.

Supplementary Figure 1. Flowchart of new predictor development.

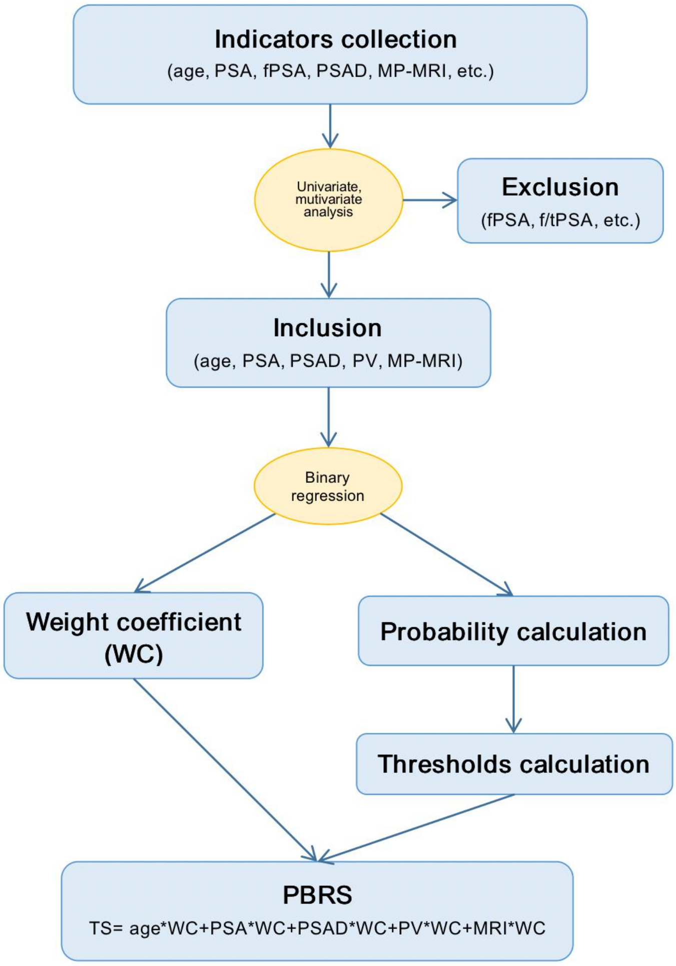

Supplement: Supplementary file 1 — Supplementary tables and figures [file 41598_2019_43427_MOESM1_ESM.pdf]
